# Protective parents and permissive children: what qualitative interviews with parents and children can tell us about the feasibility of juvenile idiopathic arthritis trials

**DOI:** 10.1186/s12969-018-0293-2

**Published:** 2018-12-04

**Authors:** Frances C. Sherratt, Louise Roper, Simon R. Stones, Flora McErlane, Matthew Peak, Michael W. Beresford, Helen Foster, Athimalaipet V. Ramanan, Madeleine Rooney, Eileen Baildam, Bridget Young

**Affiliations:** 1 Department of Psychological Sciences, University of Liverpool, Whelan Building, Brownlow Hill, Liverpool, L69 3GB UK; 2 School of Healthcare, University of Leeds, Leeds, UK; 3 Great North Children’s Hospital, Newcastle upon Tyne, UK; 4 Institute Cellular Medicine, Newcastle University, Newcastle upon Tyne, UK; 5 Clinical Research Division, Alder Hey Children’s NHS Foundation Trust, Liverpool, UK; 6 Department of Paediatric Rheumatology, Alder Hey Children’s NHS Foundation Trust, Liverpool, UK; 7 Department of Women’s and Children’s Health, Institute of Translational Medicine, University of Liverpool, Liverpool, UK; 8 University Hospitals Bristol NHS Foundation Trust & Bristol Medical School, University of Bristol, Bristol, UK; 9 School of Medicine, Dentistry and Biomedical Sciences, Queens University of Belfast, Belfast, UK

**Keywords:** Juvenile idiopathic arthritis, JIA, Randomised controlled trial, Recruitment, Corticosteroids, Qualitative, Children, Feasibility, Parents, Patients

## Abstract

**Background:**

Patient recruitment can be very challenging in paediatric studies, especially in relatively uncommon conditions, such as juvenile idiopathic arthritis (JIA). However, involving children and young people (CYP) in the design of such trials could promise a more rapid trajectory towards making evidence-based treatments available. Studies involving CYP are advocated in the literature but we are not aware of any early stage feasibility studies that have qualitatively accessed the perspectives of parents and CYP with a long term condition to inform design and conduct of a trial. In the context of a feasibility study to inform the design of a proposed randomised controlled trial of corticosteroid induction regimen in JIA, we explored families’ perspectives on the proposed trial and on JIA trials generally.

**Methods:**

We analysed interviews with 27 participants (8 CYP aged 8–16 years and 19 parents) from four UK paediatric rheumatology centres. CYP had recently received corticosteroids to treat JIA. Audio-recorded interviews were transcribed and analysed thematically, drawing on the Framework Method.

**Results:**

Both parents and CYP were capable of engaging with the logic of the proposed trial but pointed to challenges with its design. Treatment preferences influenced willingness to participate in the proposed trial. The preferences of older children and their parents often differed, with CYP being more willing to participate in the proposed trial than parents. Families’ current treatment preferences were largely informed by past positive and negative treatment experiences. Some participants also indicated that their treatment preferences were influenced by those of their clinicians.

**Conclusion:**

Previous research has typically focused on deficits in patients’ understandings of trials. We found that both parents and CYP understood trial concepts and were able to identify potential flaws in the proposed trial. We propose recommendations to optimise the design of a planned corticosteroid induction regimen trial in JIA. Accessing both parents’ and CYP’s perspectives helps to identify and address recruitment challenges, which will ultimately optimise informed consent and future recruitment.

**Electronic supplementary material:**

The online version of this article (10.1186/s12969-018-0293-2) contains supplementary material, which is available to authorized users.

## Introduction

Juvenile idiopathic arthritis (JIA) refers to a collection of inflammatory arthritides (commonly divided into several subtypes) with symptom onset at, or before the age of 16 years [1]. There has been a recent increase in the number of JIA randomised controlled trials [2]. Recruitment is crucial to the success of such trials, and poor recruitment can inflate costs [3], result in underpowered trials, and lead to potentially effective interventions being abandoned or delayed [4]. Approximately 45% of UK multi-centre trials do not achieve their original recruitment target, often resulting in costly recruitment extensions [5].

Patient recruitment can be especially challenging in paediatric studies, and in relatively uncommon conditions, such as JIA [6, 7]. Compared with trials in adult rheumatology, consent seeking can be more complex due to the need to involve both child and parents in enrolment decisions [8], and some drug treatments may be poorly tolerated by younger children [9]. Innovative trial methodology and involving parents and children and young people (CYP) in the design of JIA trials promises a more rapid trajectory towards making evidence-based treatments available to young patients [10, 11].

Increasingly, researchers are using qualitative methods to explore patients’ views of trial feasibility, and to proactively identify and address problems that may otherwise undermine a trial [12, 13]. Early stage feasibility studies, without a randomised component, are a distinct subtype of feasibility study. These are often used to resolve fundamental questions, such as which treatments to compare, or uncertainties about the ‘in principle’ acceptability of a trial to patients and recruiters before committing resources to a pilot or full randomised trial.

Although studies involving CYP are advocated in the literature [14, 15], we are aware of only one early stage feasibility study that has qualitatively investigated perspectives of both parents and CYP to inform the design and conduct of a trial [14]. This recent study focused on the experiences of CYP with acute osteomyelitis or septic arthritis, whereas the current study explored the perspectives of CYP with JIA. JIA is a long term condition, CYP with JIA may find the prospect of trial participation especially challenging due to preferences arising from their ongoing experiences of the condition and different treatments [15]. Research is needed to shed light on such issues. This article explores what an early phase qualitative feasibility study, involving interviews with both parents and CYP, can tell us about the viability of a proposed JIA trial of corticosteroid induction regimens. We also report on the wider barriers and opportunities for recruitment in JIA trials.

## Methods

We adopted a qualitative approach involving semi-structured interviews. Qualitative research provides in-depth insights on participants’ experiences and perspectives. Such studies are typically characterised by smaller sample sizes but rich, voluminous data [16]. Qualitative research has been used to identify and address key uncertainties in planning and designing proposed trials for adult patients [12]. Semi-structured interviews are a qualitative research method well suited to exploring the perceptions of patients regarding complex and sensitive issues [17], as these enable families to raise matters of importance to them, and for interviewers to inform families about trials and clarify any misunderstandings.

A Research Ethics Committee in the North East of England (The North East – Newcastle and North Tyneside 2) approved this study (16/NE/0047).

### Participant selection

Within four UK paediatric and adolescent rheumatology centres, clinicians approached families of children and young people who met the eligibility criteria (Table 1), briefly described the study and requested verbal permission for a researcher to contact families. Children and young people had a confirmed diagnosis of JIA in line with current guidance [1, 18]. FS and LR, both experienced in qualitative research in health settings, conducted the interviews after seeking informed consent from parents and assent or consent from CYPs. Interviews took place from August 2016 to March 2017 and were audio recorded and transcribed. The research team monitored sampling characteristics to ensure these were inclusive of patients of varying ages, and of families from a range of treatment centres and socioeconomic backgrounds. Sampling for interviews ceased when data saturation was reached, that is, when further interviews were no longer contributing new information [19].

**Table 1:** Inclusion criteria for qualitative interviews with families

Inclusion criteria • CYP ≤ 16 years. • CYP has received a clinical diagnosis of JIA. • CYP has recent experience (≤ 12 months) of at least one of four corticosteroid delivery routes: (a) oral prednisolone (tablets); (b) intravenous methylprednisolone (IV); (c) intra-articular injection(s) (IACI), or; (d) intra-muscular injection (IM).	

### Interview protocol

Semi-structured, topic-guided interviews (Additional file 1: Appendix A) were conversational to allow participants to raise and reflect on matters of concern to them. While we interviewed all parents regardless of their child’s age, only children aged eight years and over were interviewed. We developed separate topic guides for parents, children (8–12 years), and young people (13–16 years), and adapted these throughout the study, guided by the ongoing analysis. We also developed stimulus materials to facilitate the interviews, including a video, a flow chart illustrating the proposed trial, and prompt cards detailing potential benefits and side effects of the four corticosteroid delivery routes (Additional file 2: Appendix B). Consultant paediatric rheumatologists advised on the development of the prompt cards. These cards listed key points about the delivery routes that the rheumatologists perceived relevant to patients and families. The cards were intended to facilitate discussion in the interviews, rather than to provide an exhaustive list of benefits and side effects of the delivery routes.

In brief, the proposed trial was described to participants as comparing four corticosteroid delivery routes (hereon referred to as treatments): (a) oral prednisolone (referred to herein as tablets); (b) intravenous methylprednisolone (IV); (c) intra-articular injection(s) (IACI), and; (d) intra-muscular injection (IM). These four treatments are widely used in clinical practice for JIA, although the only informative evidence-base of effectiveness and efficacy is for IACI [20, 21]. Referring to the prompt cards the interviewer described the four delivery routes to families, including the process of delivery (e.g. duration) and potential pros and cons. While a trial could help to establish which of the treatments is most effective in treating JIA, the feasibility of such a trial is uncertain.

### Qualitative analysis

We drew on The Framework Method [16], an approach to the thematic analysis of qualitative of data [22], and used NVivo 10 to assist with data indexing and coding [23]. FS, LR and BY initially read a selection of transcripts and discussed the developing analysis. FS and LR double-coded approximately 10% of transcripts, discussing divergences and their resolution, leading to the development of a preliminary coding framework. Remaining transcripts were coded by either FS or LR and further analysis meetings were organised involving the research team (BY, LR, and FS) throughout the course of analysis to discuss convergences and divergences, identify quotes for report writing, and review data saturation.

In quantitative research, numbers contribute to the persuasive “power” of the findings, whereas, in qualitative research, the words of participants enable the reader to judge whether the research team’s interpretations of the data are grounded in the experience of the participants [24]. The research team selected participant quotes for this report to illuminate and explicate the researchers’ synopsis of the themes identified through systematic data analysis [25, 26]. A draft report of the analysis containing extensive data extracts was circulated to the wider study team. This enabled investigator triangulation helping to ‘test’ and refine the analysis. Participants were sent a summary of the study findings.

## Results

### Participants

All 26 eligible families identified by clinicians agreed to researcher contact. Of these, nine could not be contacted and two declined. Twenty-eight participants from 15 families completed or attempted an interview (58% response rate), comprising nine patients and 19 parents (Table 2). In one of the 28 interviews, we were unable to sufficiently engage the patient (C8) in discussing the concepts that we wished to explore. As no meaningful data were obtained this patient’s interview was not transcribed or analysed, although his parents’ interview was. Another patient’s interview (C11) was unusually short (six minutes) as the family had limited time but her parent was interviewed and child and parent interviews were analysed. Excluding the outlier interview (C11), interviews lasted from 23 to 76 min (Media*n* = 43 [IQR = 35–54] minutes).

**Table 2:** Characteristics of participants in the study

Family number	Parent interviewed	Child/young person’s age	Child/young person’s JIA status (reported by clinician)	Treatments experienced by the child/young person^b^			
				Tablets	IV	IM	IACI
1 ^a^	Mo, Fa	11–13	Flare	✓	✓	✓	✓
2	Mo	5–7	Flare	✓			✓
3 ^a^	Mo	14–16	Flare	✓	✓		✓
4^c^	Mo, Fa	1–4, 5–7	Diagnosis, Flare	✓			✓
5	Mo	1–4	Diagnosis				✓
6	Mo	5–7	Diagnosis	✓			✓
7	Mo, Fa	1–4	Diagnosis				✓
8 ^a^	Mo, Fa	8–10	Flare	✓			✓
9 ^a^	Mo	11–13	Diagnosis		✓		✓
10 ^a^	Mo	14–16	Flare	✓	✓	✓	✓
11 ^a^	Mo	8–10	Flare	✓			✓
12 ^a^	Mo	11–13	Diagnosis	✓	✓		
13 ^a^	Mo	14–16	Flare		✓		✓
14 ^a^	Mo	14–16	Flare			✓	✓
15	Fa	14–16	Flare	✓	✓		

*Mo* = Mother, *Fa* = Father, Tablets = Oral prednisolone, *IV* = Intravenous methylprednisolone, *IM* = Intra-muscular injection, *IACI* = Intra-articular injection(s). JIA status as reported by clinician

^a^Families where the child/young person was also interviewed, ^b^Reported by parents, ^c^Family with two children with JIA

Participants were interviewed in their homes (*n* = 9), in a private setting in the paediatric rheumatology clinic (*n* = 4), in a parent’s workplace (*n* = 1) or by telephone (*n* = 1). Of the nine CYP, two were interviewed jointly with their parents, while seven were interviewed separately. Of 19 parents, 14 were interviewed separately while five were interviewed with their child present, including the two noted above, and three parents whose children were ≤ 7 years and ineligible for interview.

As Table 2 shows, CYP’s ages ranged from 1 to 16 years, and most were female (*n* = 9/16, 56%). One family had two CYP with JIA. All but two families (*n* = 13/15) had experienced at least two treatments, and most CYP had established JIA with a recent disease flare (*n* = 10/16). IACI was the most common treatment experienced (*n* = 14/16). While the inclusion criteria stated that patients needed to have had corticosteroids within the past 12 months (see Table 1), all families reported that patients were treated within the past 6 months and almost a third (*n* = 4/15) were actively receiving at least one corticosteroid treatment at the time of interview. We obtained families’ postcodes wherever possible and used the Indices of Multiple Deprivation (IMD), a measure of socio-economic status, to inform sampling and ensure representation from all socio-economic groups: 6/13 (46%) families lived in areas of high deprivation (IMD decile 1–3), 5/13 (38%) in areas of moderate deprivation (IMD decile 4–6), and 2/13 (15%) in the least deprived (IMD decile 7–10) areas of England [27].

### Willingness to participate: Protective parents and permissive children

Participant is indicated (Mo = mother, Fa = father, or C = child) with family number (see Table 2 for family number reference). Patients’ age categories are provided (e.g. 14-16y). Ellipsis (...) indicate omitted text and square brackets indicate explanatory text.

Families recognised the need for JIA trials and some expressed enthusiasm for the proposed trial: “*I would [participate] ‘cause I’ve always been up for like helping with research* (Mo2_5-7y)”; “*It is a very good topic to actually do a research on […] it’s one that’s needed because there are many ways of [delivering] steroids*” (C3_14-16y). CYP often suggested that participation in the proposed trial could be of benefit to either themselves or others: “*I’d try it [the trial], yeah… It seems fine… If it’s going to help, it’s going to help*” (C14_14-16y).

How CYP and parents perceived randomisation influenced their views of the trial. For the most part, both parties appeared to grasp the rationale for randomisation as a means to avoid bias: “*I do think it will be best if it’s chosen by a computer because some patients might be biased*.” (C3_14-16y). However, at times some parents struggled to reconcile randomisation with a need to trust in clinicians’ expertise: “*I’d go with just whatever’s the best one and that’s why, that’s why I’m trusting [the doctors] to get her better… I wouldn’t be comfortable with the computer randomly selecting what would be the best course of treatment for her*.” (Fa15_14-16y).

Others struggled to reconcile randomisation with their need as parents to have confidence in the care being provided for their child: “*We were told the steroid injections would help her, so that sort of put confidence in us. But for someone to say we’re not quite sure which one the best one is, so we’re going to do it randomly, I think that would put a lot of sort of discomfort in us*.” (Mo7_1-4y). In contrast, only one young person was opposed to participating in the proposed trial, rejecting randomisation in favour of a clinician-informed treatment: “*I would have said no because... the doctor needs to tell you what’s best for you and not a computer because a computer doesn’t know that*.” (C12_11-13y).

The comments of other CYP indicated they were open to participating and some expressed a degree of nonchalance to their treatment being randomised as part of research participation: “*I’d be like ‘It’s been happening for this long, I don’t care anymore, you can do whatever, do anything with my body, it’s alright!’*” (C13_14-16y). In some of the joint interviews, CYP were more permissive about research than their parents and suggested that they would be more willing to participate in such a trial:

- C13: *[At diagnosis] I haven’t experienced any of them [treatments] so far so it wouldn’t hurt to try any of them…*
- Mo13: *I think I might have been a little bit more concerned ‘cause I think I would want, at the beginning when we didn’t know a lot about it, I think I would have been thinking, oh no, I need to have the consultant say what’s best for him.* (14-16y)

This permissive attitude among CYP may have been founded in part on feeling confident that clinicians would always prioritise their care over the trial: “*I’d probably take part in it just to like help the research and, ‘cause I think if something didn’t work on me I know that… they’d just put me on something else like in the end*.” (C9_11-13y).

### Barriers and facilitators to participation

Our analysis identified several potential recruitment barriers and facilitators to the proposed trial, some of which were voiced by parents but not CYP and vice versa. For example, some parents questioned or remarked on the number of visits to clinic that the proposed trial would entail, noting that it was already challenging to attend clinic appointments due to work or school commitments. Parents thereby implied that their willingness to participate would hinge on the convenience or otherwise of the trial: “*Would that entail, mean more hospital visits though, to do this trial? ‘Cause obviously with work we’d be struggling, it depends how often that would be.*” (Mo2_5-7y). Some CYP acknowledged these factors but did not suggest that this would be a barrier to participation: “*Mam doesn’t really mind going to the hospital and that, because obviously she does it because it helps me.*” (C14_14-16y).

#### Past treatment experiences

Families spoke of their experience of different treatments over numerous disease flares since diagnosis. This experience heavily influenced their views on the proposed trial and their willingness to participate. Reflecting on a treatment that “*had worked*”, one mother indicated that she would like her child to receive the same treatment if they experienced a future flare-up: “*When you’re first diagnosed you will try any of the medication, but now we know what works for [my child] I would want to stick with that*.” (Mo14_14-16y). Conversely, on realising that joining the trial could mean her child receiving a treatment that had previously “*not worked*”, another mother questioned the logic of offering the same treatment in the context of a trial: “*If it’s not worked this time, what makes them think it’s going to work the second time?*” (Mo6_5-7y). That is, parents struggled to see the logic behind randomising a child to a treatment that they had previously experienced as ineffective, and this was an important issue they would want clinicians to address should such a trial be offered to them.

Older children and their parents frequently differed in their treatment preferences, with both parties often noting drawbacks of treatments that the other had not identified:

- C12: *I’d rather [have IV].*
- Mo12: *You’d rather have [IV] than just have a needle in your bum? I think I’d rather have the needle in my bum than be sat here.*
- C12: *Wouldn’t [IM] hurt? That would hurt more surely?*
- Mo12: *… well you’ve had to come here for three days [for IV].*
- C12: *It doesn’t bother me. It’s like an hour on this and then it’s done.* (11-13y)
- Mo13: *… to me, [IM] would be a lot less painful going in your butt ‘cause you’ve got that little meatiness, do you know, something to put it in.*
- C13: *Any muscle anywhere else I probably would have been fine. Like you could just stick it in my thigh or something that’s okay but I don’t want to have to get the moon out for you.* (14-16y)

Some CYP were receiving corticosteroids at the time they were interviewed. Despite being unable to assess the effect of the current treatment in reducing symptoms at that point in time, one young person explained that he preferred his current treatment over other treatments, even ones he had not been prescribed: “*I’d say [I prefer] this one… because my experience right now… I feel quite, quite fine on this. Probably [would then have] tablets since I haven’t experienced those [tablets]*” (C13_14-16y), implying that treatment side effects also weighed heavily in his treatment preferences.

CYP with multiple affected joints often received corticosteroids via several delivery routes, either simultaneously or consecutively, which made it difficult for them and their parents to differentiate the effect of an individual treatment: “*I don’t know [which is best] because like I feel like the intravenous and the tablets go along with each other in a way because I’m on both at the minute.*” (C12_11-13y). Most families also had experience of non-corticosteroid treatments for JIA, such as other immunosuppressive medication, hydrotherapy, or physiotherapy, and some were concerned about the possibility that interactions between corticosteroid and non-corticosteroid treatments could jeopardise the validity of the proposed trial’s findings if non-corticosteroid treatment effects were not controlled for: “*The challenge is that they don’t have this treatment independently of other things, do they? It’s always combined with something else or it has been in our experience. So, it’ll be steroids plus something else*.” (Mo10 14-16y). Some parents also queried whether they could continue with existing treatments if their child was to participate in a trial of corticosteroids: “*I don’t know, would they stop the methotrexate or would they just carry it on?*” (Mo4 1-4y, 5-7y). These illustrate further issues that clinicians would need to address when inviting families to take part in JIA trials.

#### Perceived treatment suitability

While families did not view any one of the four delivery routes as unsuitable overall to be evaluated in the proposed trial, they did perceive certain treatments as more or less acceptable for certain CYP. Families held these views even when they did not have direct experience of the treatment concerned. Parents believed that treatment suitability depended on a child’s age. For example, a mother anticipated that her eight-year-old would not tolerate IV: “*He’s not going to want to stand there for hours… I would imagine if he got lots and lots of flare-ups, and he is older and he understands it, I can see the logic behind that*.” (Mo11 8-10y). While a child’s age seemed important to parents in making decisions about treatments, and therefore in designing the trial, CYP did not comment on this.

Both parents and CYP believed that treatments varied in their suitability for different patient groups or JIA subtypes. For example, one child suggested that IV is more suitable when a child has more than one joint affected: “*If you have a flare and it doesn’t just affect one joint, then [IV], it’d kind of help you more.*” (C9_ 11-13y). Similarly, a mother of a child with localised JIA echoed the view that treatments varied in their suitability for different JIA subtypes and implied that IV could also be potentially harmful for her child due to its systemic nature: “*The intravenous one would affect the whole body, so I don’t know whether that would be a good thing or not. I’ve never experienced that, we’ve only ever had the joint injections*.” (Mo2 5-7y). Parents and some children therefore struggled to see the logic of a trial in which they might receive a treatment, that, based on their knowledge of JIA and how treatments worked, was ill suited to their subtype of JIA.

### Being approached about research at diagnosis

Given the powerful influence of past treatment experiences on current treatment preferences, it was unsurprising that most participants were more comfortable with the idea of participating in the trial if invited at diagnosis when treatments would be new to families: “*[We would participate] at the beginning when he had it, probably it will be different but now, no way… we had such a big, big problems with his injection…*” (Mo8_ 8-10y). Nevertheless, there were some exceptions. Several parents recalled the emotional upheaval of learning that their child had a life changing condition and their strong desire for certainty at that time. One mother explained that if she was asked to enter her child into a trial in the aftermath of diagnosis: “*I’d probably say ‘Not yet’*” (Mo9_ 11-13y).

CYP did not view trial recruitment at diagnosis to be problematic, again pointing to divergence in the views of the two parties. Overall, parents often described their children as resilient and CYP themselves rarely described the emotional upheaval of receiving a diagnosis but they did reflect on how the diagnosis had affected their parents:

- I: *What was it like being told about having arthritis?*
- C9: *I don’t know, I think I took it a bit better than my mum, ‘cause mums are mums, so they get a bit worried.* (11-13y)

#### Families’ accounts of clinicians’ treatment preferences

Both parents and CYP referred to the relationships they had with their clinician and how they trusted them to accurately inform them about treatments and provide them with optimal care: “*The doctors have been so good… I’ve got to trust what they are telling me and what they think is best*” (Fa15_ 14-16y).

- I: *Who do you think would be the best person to give you more information about the research study?*
- C1: *Probably doctors and that so you know it’s true, in case like random people just say this works.* (11-13y)

Families spoke about treatment preferences that their clinician had voiced when making recommendations about treatment during routine consultations and that clinicians’ preferences too depended on a child’s age, medical history and JIA subtype: “*And age thing as well, and how long they’ve had [JIA]. Because [the doctor] tends to do it with their age doesn’t she?*” (Mo1_ 11-13y). Participants indicated that clinician treatment preferences influenced their perception of treatment efficacy and suitability: “*Probably the joint injections [would be best] ‘cause [the doctor] said they work better ‘cause it’s straight into the joint*.” (C9_ 11-13y).

## Discussion

This is the first, pre-trial qualitative feasibility study that has involved both parents and CYP with a long term condition. Although studies involving CYP are advocated in the literature [14, 15], we are aware of only one other early stage feasibility study that qualitatively investigated both parents’ and CYP’s input in trial design and conduct [14]. This explored families’ views on the feasibility of a trial to determine optimum duration of intravenous antibiotic therapy for children with acute osteomyelitis or septic arthritis The current study recruited CYP with a long term condition, for whom trial participation can be especially challenging [15]. While much research has focused on deficits in patients’ understanding of trials [4], we found that parents and CYP were able to engage with the logic of the proposed trial and identify potential flaws in its design, despite the scenario being hypothetical [28, 29]. This suggests that pre-trial, qualitative feasibility studies with families can inform and optimise trial design, thereby aiming to avoid common recruitment pitfalls [3, 4], by drawing on the in-depth knowledge and insight families acquire in coping with a long term condition.

Contrary to previous research [30], we found that parents and CYP frequently differed in their willingness to participate in the proposed trial and in their treatment preferences. While CYP were more permissive towards the trial than parents, they sometimes identified concerns that parents did not raise. This also echoes a previous pre-trial feasibility study which found that patients with acute osteomyelitis or septic arthritis often downplayed the impact of the illness or focused on different issues to their parents [14]. Previous research has found that parents often adopt an executive or managerial role in their child’s treatment and care [31], working to identify, anticipate and meet their child’s needs [32]. Based on this previous work and findings from the current study, we propose that CYP’s permissiveness reflects their reliance upon their parents to protect them from harm and coordinate their care. CYP also trusted clinicians with their care and, unlike parents, they did not identify trial inconveniences (e.g. travel), which may have also contributed to their permissive orientation. Current guidance encourages recruiters to support families in sharing decisions regarding research [14]. Given that our study and others show that CYP can offer valuable input to trial design [14], the viewpoints of both parents and CYP are likely to be valuable in informing and improving future paediatric clinical trials and clinical practice. The findings have helped us in developing recommendations for a proposed corticosteroid induction regimen trial (Table 3). Several of these recommendations will be useful in developing future trials in JIA more broadly and in avoiding common recruitment pitfalls.

**Table 3:** Summary of the recommendations for the design of a future corticosteroid induction regimen trial in JIA

1. The views of parents and children and young people are important in informing trial design and conduct. 2. Although families may not deem one treatment as unsuitable overall, trialists need to establish whether *all* treatments are suitable for *all* patients with JIA. If this is not possible, amendments to the eligibility criteria or fewer treatments will be needed. 3. Further qualitative work should explore clinicians’ views on a future corticosteroid induction regimen trial in JIA, to establish clinical equipoise and support for the trial. 4. Trialists should consider methods of making a future trial more accessible to parents and children and young people, such as combining trial follow-up assessments with clinic appointments.	

While families did not deem any single treatment as unsuitable to evaluate in the proposed trial, they believed that treatments needed to be tailored to CYP depending on factors such as age and JIA subtype. Previous experience of treatments also influenced such beliefs. Our findings indicate that where families have treatment preferences that are experience-based (e.g. trials evaluating established treatments), rather than anticipated (e.g. trials of treatments that are new to families), recruitment and randomisation is likely to be especially challenging [33–36]. This is pertinent as CYP with JIA receive treatments on repeated occasions [21]. Clinicians recruiting to such trials will need to be prepared to respond to questions from families who have previously experienced a particular treatment to be ineffective. Trialists should consider which treatment options can be evaluated in a trial and will be tolerated by CYP with JIA. Some trials may need to adapt the eligibility criteria or the treatments included to make the trial more acceptable to families. It is also currently unclear how decision-making about trial participation is managed within families when treatment preferences conflict; further research would be beneficial.

Families described how clinicians had shaped their treatment preferences via previous discussions about the suitability of different treatments, based on factors, such as a child’s age and the number of joints affected. Such discussions are part of good care in routine clinical practice, but equally, when invited to consider participation in a trial, families cannot be expected to put aside knowledge they have previously gleaned from clinicians. While clinician and patient treatment preferences are often regarded as separate entities, our findings indicate that the two can be intertwined and that the influence of previous clinician-family discussions about treatments needs to be considered when communicating with families about trials. For example, clinicians will need to respond to families’ concerns about using a treatment that clinicians may have previously told them was unsuitable. Indeed, clinicians and their treatment preferences can often be a considerable barrier to patient participation in paediatric trials [37, 38]. Further qualitative work exploring clinicians’ views of future JIA trials would help to address such potential difficulties.

Similar to previous research [39], we found that trial inconvenience, such as additional appointments, will also likely be a recruitment barrier for parents. Trialists will need to explore options to reduce burden, such as completing follow-up appointments by telephone or Skype, and arranging appointments at participants’ convenience (e.g. out of school hours).

We interviewed socio-economically diverse families from across England with children of different ages and varied treatment experiences**.** While we had interview data from only one patient aged under 10 years, our sample did include parents of children aged 1–16 years. Additionally, we interviewed families of patients who had different sub-types of JIA and numbers of affected joints, although we did not actively sample for these clinical characteristics. In accessing the perspectives of CYP our approach is consistent with current guidance on including the voices of children in research [14]. Furthermore, the competence of chronically ill children in communicating about health related matters can often exceed their chronological age [40]. We did not interview families with a JIA diagnosis who had not yet been treated with corticosteroids, and we acknowledge that the views of such families may differ markedly from our findings. However, interviewing families at diagnosis, but before corticosteroid treatment, would likely have been challenging, particularly given the difficulties families experience at this time [41], which we also reported here. This study’s sample size is typical and appropriate for a qualitative study given that the inferences drawn are not about prevalence or statistical distribution [16]. Rather, our inferences concern the nature of families’ perspectives and the value of these in informing the design of JIA trials. Similar to deliberative engagement methods [42], we used stimulus materials (see Additional file 2: Appendix B) and discursive interviews to enable families to raise matters of importance to them, and for us to inform families about trials and clarify any key misunderstandings.

## Conclusion

This pre-trial qualitative feasibility study demonstrated that families could engage in the logic of a proposed trial in JIA and provide valuable input into trial design before further investment of resources. We identified potential barriers to recruitment for a corticosteroid induction regimen trial in JIA, divergent views between parents and CYP and areas for further exploration, including clinician treatment preferences. This study highlights the importance of including families in pre-trial feasibility work to illuminate barriers to recruitment and to inform strategies to improve informed consent and recruitment.

## Additional files

Additional file 1: **Appendix A.** Topic guide for parents’ qualitative interviews. (DOCX 28 kb)

Additional file 2: **Appendix B.** Stimulus. (PUB 1131 kb)

## Abbreviations

C: Child
CYP: Children and young people
Fa: Father
HTA: Health Technology Assessment
IACI: Intra-articular injection(s)
IM: Intra-muscular injection
IMD: Indices of Multiple Deprivation
IV: Intravenous methylprednisolone
JIA: Juvenile Idiopathic Arthritis
Mo: Mother
NIHR: National Institute for Health Research
SIRJIA: Steroid Induction Regimen for Juvenile Idiopathic Arthritis
