# Supplementary material for: Protective parents and permissive children: what qualitative interviews with parents and children can tell us about the feasibility of juvenile idiopathic arthritis trials
Source: Pediatr Rheumatol Online J. 2018 Dec 4;16:76. doi: 10.1186/s12969-018-0293-2 (PMC6278083; doi:10.1186/s12969-018-0293-2)
Supplement: Supplementary file 1 — Appendix A. Topic guide for parents’ qualitative interviews. (DOCX 28 kb) [file 12969_2018_293_MOESM1_ESM.docx]

Topic guide for parents’ qualitative interviews

**Families’ experiences of corticosteroid treatments in children and young people with juvenile idiopathic arthritis (JIA)**

NOTE: Qualitative interviews will be conversational and tailored to the individual participant. So, this is only an outline of the topics and questions that will be covered and it will be updated in the light of the ongoing interviews and analysis.

***First section***

*This first bit of the interview is all about YOUR child and arthritis.*

- What it was like for your child (always use child’s first name as appropriate) when s/he first started having problems with his/her joints?
- What problems was s/he having?
- Did it take long to find out what was wrong?

***Second section***

*This part of the interview is to ask what YOU think about the diagnosis and how it was explained it to you*.

- How was the arthritis diagnosed? (tests etc? )
- How did the doctors explain about arthritis?
- What was it like to learn your child had arthritis? What did you understand about the short /longer term impacts of arthritis?
- What treatments did the doctor talk about at first? (Did they talk to your child as well as you?)
- What did you feel about the treatments/medicines when you first heard about them?
- What do you think would have happened without the treatment?

***Third section***

*In this part I would like to ask you what it was like for your child to have the different types of treatment for arthritis.*

- What treatments/medicines did your child have after the arthritis was first diagnosed? PROMPT for different types of treatment?
- How was it decided that s/he would have that sort of treatment? Did the doctor recommend a treatment? Did you feel that you had a say?
- What were the different treatments for? PROMPT about steroids?
- What was it like for your child to have those/that sort of steroid treatment? How s/he did you feel before/during/after?

*One of the types of medicine that your child was given is called steroids. Doctors gave steroids to people in different ways/routes. This part of the interview is about those different ways.*

- You’ve explained that your child had steroids by x route(explain)
- What was it like for them having to have steroids this way?
- Can you say what were the good aspects? What wasn’t good about it? **PROMPT**: Taste? Hospital stay? Injection? Pain etc?
- This is another way of giving steroids (show picture/prop of another route of delivery)?
- What do you think would be good /bad about this one? **PROMPT**: for different pros and cons (repeat for all delivery routes)
- Which way do you think is best for your child?
- PROBE What makes you say that? Which do you think is best in the longer term?
- Which do you think the doctor /your child is best? PROMPT explore reasons
- That was x months ago - how is your child getting on now with the arthritis? Explore how things changed in days, weeks after steroid treatment?
- Does your child/ the doctor think things are better now? Why?

**Fourth section**

*In this final section, I would like to ask you what you think about the idea of a medical study that doctors are planning to carry out, to find out the very best way of giving steroid treatment for young people who have arthritis. As your child has already had X your opinions would be very helpful to us.*

Here is a picture which explains what happens when a young person is asked whether they want to take part in a medical study. SHOW PICTURE OF PROCESS/ DESIGN FOR RCT 13+ YRS and talk through it (following is just an outline summary).

First of all the medical study is explained to the young person and their family. So, a doctor might explain it like this… . Then the young person will have time to think whether they want to take part or not. If they don’t want to take part that is absolutely fine. If they do want to take part, then a computer decides WHICH way the steroid treatment is given. So, the main difference in this medical study is to do with the different ways of giving the steriod treatments shown in the picture (name and explain the different options/ways of steroid delivery). All children have the same type of medicine –steroids -but it is given in different ways.

- What sort of things came in to your mind as you heard me describe the medical study?
- Do you have any initial questions or comments about the medical study?

Would you mind having a read through this brief information sheet and then I’ll ask you a few questions about it. Show sheet about planned trial.

- Is there anything you don’t understand?
- Which parts are interesting to you?
- What would you like to know more about?
- How would you feel about a computer (not doctor/parent/you) deciding the way that the steroid treatment is given?
- Are any of the treatments better than the others? PROMPT about what makes parent think that?
- Would you be inclined to say ‘no’ to any of the ways of treatments? What about other parents of children with arthritis, do you think they might say no to any of these?
- Looking back to the days after your child was first diagnosed with arthritis, do you think would you have agreed to take part in a study like this? PROMPT explore reasons? If no, what might make the study more acceptable?
- *(Explain/reassure afterwards, as necessary, that this is only a planned medical study at the moment. Not actually requesting participation.)*
- We were talking earlier about the pros and cons of the different ways/routes of having the steroids… . Just to finish, this card shows the ‘clinical’ advantages and disadvantages of these different ways/routes. SHOW CARD.
  1. Which of these (if any) particularly strike you? Which ones would be important to you, what about your child?
  2. Having been made aware of this information, would you be willing for you child to take part in this future medical study? *Probe reasons for any changes in/maintenance of original decision. (Again explain/reassure afterwards, as necessary, that this is only a theoretical question. Not actually requesting participation.)*
